# Spiro­[cyclo­pentane-1,2′(1′*H*)-pyrido[2,3-*d*]pyrimidin]-4′(3′*H*)-one

**DOI:** 10.1107/S1600536810027479

**Published:** 2010-08-18

**Authors:** Daxin Shi, Liupan Yang, Jianhong Tang, Xiuzhen Wang, Jiarong Li

**Affiliations:** a School of Chemical Engineering and environment, Beijing Institue of Technology, Beijing 100081, People’s Republic of China

## Abstract

The title compound, C_11_H_13_N_2_O, was obtained by cyclo­condensation of 2-amino­pyridine-3-carbonitrile with cyclo­penta­none. The mol­ecule is built up from two fused six-membered rings and one five-membered ring linked through a spiro C atom. Both the pyrimidine and the cyclo­pentane rings adopt envelope conformations. In the crystal structure, mol­ecules are linked by inter­molecular N—H⋯O hydrogen bonds.

## Related literature

Many compounds containing the pyrido[2,3-d]pyrimidine scaffold show pharmacological properties such as anti­tumor (Gangjee *et al.*, 1996 ▶), analgesic (Cordeu *et al.*, 2007 ▶) and anti­bacterial (Robins & Hitchings, 1958 ▶) activities. 2-Substituted 2,3-dihydro­pyrido[2,3-*d*]pyrimidin-4(1*H*)-one derivatives can be obtained by a Friedlander quinoline condensation, see: Li *et al.* (2008 ▶). For a related structure, see: Zhang *et al.* (2008 ▶). For our previous work, see: Li *et al.* (2009 ▶); Ma *et al.* (2006 ▶).
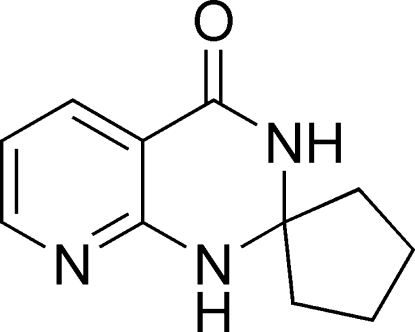


## Experimental

### 

#### Crystal data

C_11_H_13_N_3_O
*M* *_r_* = 203.24
Orthorhombic, 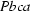

*a* = 10.400 (1) Å
*b* = 12.1650 (15) Å
*c* = 15.370 (2) Å
*V* = 1944.6 (4) Å^3^
*Z* = 8
Mo *K*α radiation
μ = 0.09 mm^−1^
*T* = 113 K
0.32 × 0.30 × 0.28 mm

#### Data collection

Rigaku Saturn724 CCD diffractometer
Absorption correction: multi-scan (*Crystal Clear-SM Expert*; Rigaku/MSC, 2009 ▶) *T*_min_ = 0.971, *T*_max_ = 0.974
21571 measured reflections
2314 independent reflections
2168 reflections with *I* > 2σ(*I*)
*R*_int_ = 0.037

#### Refinement

*R*[*F* ^2^ > 2σ(*F* ^2^)] = 0.040
*wR*(*F* ^2^) = 0.103
*S* = 1.05
2314 reflections
144 parameters
H atoms treated by a mixture of independent and constrained refinement
Δρ_max_ = 0.40 e Å^−3^
Δρ_min_ = −0.20 e Å^−3^

### 

Data collection: *Crystal Clear-SM Expert* (Rigaku/MSC, 2009 ▶); cell refinement: *Crystal Clear-SM Expert*; data reduction: *Crystal Clear-SM Expert*; program(s) used to solve structure: *SHELXS97* (Sheldrick, 2008 ▶); program(s) used to refine structure: *SHELXL97* (Sheldrick, 2008 ▶); molecular graphics: *CrystalStructure* (Rigaku/MSC, 2009 ▶); software used to prepare material for publication: *CrystalStructure*.

## Supplementary Material

Crystal structure: contains datablocks global, I. DOI: 10.1107/S1600536810027479/lx2156sup1.cif

Structure factors: contains datablocks I. DOI: 10.1107/S1600536810027479/lx2156Isup2.hkl

Additional supplementary materials:  crystallographic information; 3D view; checkCIF report

## Figures and Tables

**Table 1:** Hydrogen-bond geometry (Å, °)

*D*—H⋯*A*	*D*—H	H⋯*A*	*D*⋯*A*	*D*—H⋯*A*
N2—H1⋯O1^i^	0.88 (2)	2.05 (2)	2.918 (1)	170 (2)
N3—H2⋯O1^ii^	0.89 (2)	2.00 (2)	2.876 (1)	172 (1)

Symmetry codes: (i) 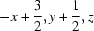; (ii) 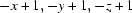.

**Fig. 1.**
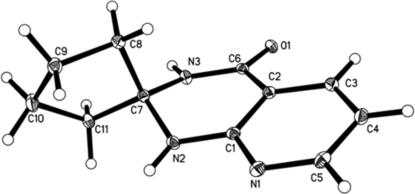
The molecular structure of the title compound with the atom numbering scheme. Displacement ellipsoids are drawn at the 30% probability level. H atoms are presented as a small cycles of arbitrary radius.

**Fig. 2.**
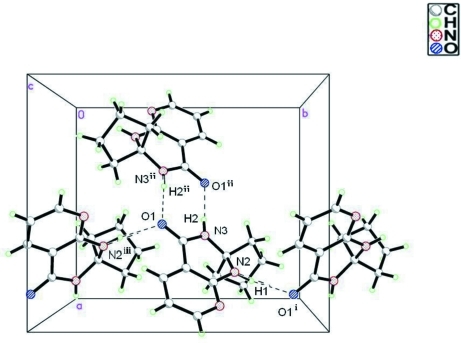
N—H···O interactions (dotted lines) in the crystal structure of the title compound. [Symmetry codes: (i) - x + 3/2, y +1/2, z; (ii) - x + 1, - y + 1, - z + 1; (iii) - x + 3/2, y - 1/2 , z.]

## supplementary crystallographic information

### Comment

Many compounds containing pyrido[2,3-d]pyrimidine scaffold show interesting pharmacological properties such as antitumor (Gangjee *et al.*, 1996), analgesic (Cordeu *et al.*, 2007) and antibacterial (Robins *et al.*, 1958) activities. 2-Substituted 2,3-dihydropyrido[2,3-d]pyrimidin-4(1*H*)-one derivatives can be obtained from the new conversion (PDF) existing in the normal Friedlander quinoline condensation (Li *et al.*, 2008). Here, we report the crystal structure of the title compound (Fig. 1).

The molecular structure (Fig. 1) is built up with two fused six-membered ring and one five-membered ring linked through a spiro C atom. The pyrimidine ring has an envelope conformation with a mean deviation of 0.1321 Å from the plane and N3 at the flap. The five-membered ring also displays an envelope conformation with a mean deviation of 0.1633 Å from the plane and atom C8 at the flap position. The geometry of the fused rings compares well with the related spiro[cyclopentane-1,2'(1'H)-quinazolin-4'(3'H)-one] (Zhang *et al.*, 2008). The crystal packing (Fig. 2) is stabilized by intermolecular N—H···O hydrogen bonds between the two N—H groups and the ketone O atoms of the neighbouring molecules (Table 1).

### Experimental

A solution of 2-amino-3-cyanopyridine (2 mmol) and sodium methylate (0.6 mmol) was refluxed in cyclopentanone (3 ml) for 1.5 h. The reaction mixture was cooled to room temperature and then filtered to give the title compound. The product was recrystallizated from a mixed solvent (ethanol:THF/1:1)to give colorless crystalline powder. M.p. 527–528 K. Spectral data: IR (KBr): 3271, 3168, 2922, 1644, 1600, 1420 cm^-1^; ^1^H NMR (DMSO,p.p.m.): 1.67–1.83 (8*H*, s, C_4_H_8_), 6.65–3.69 (1*H*, m, J = 12 Hz, ArH), 7.61 (1*H*, s, NH), 7.85–7.88 (1*H*, d, J = 7.2 Hz, ArH), 8.127(1*H*, s, NH), 8.305 (1*H*, s, ArH); ESI-MS m/*z*: [*M*+H]^+^ 204.1, [*M*+Na]^+^ 226.1; C_11_H_13_N_3_O:calcd. C 65.01, H 6.45, N 20.68; found C 65.06, H 6.47, N 20.50.

### Refinement

C—H were included in the riding model approximation with C—H distances 0.95–0.99 Å, and with *U*_iso_(H)=1.2*U*_eq_(C) or 1.5*U*_eq_(*C*)(methyl). H atoms of NH group were located in difference Fourrier maps with N—H distances 0.891–0.901 Å with *U*_iso_(H)=1.2*U*_eq_(N).

### Crystal data

**Table:**

C_11_H_13_N_3_O	*F*(000) = 864
*M**_r_* = 203.24	*D*_x_ = 1.388 Mg m^−^^3^
Orthorhombic, *P**b**c**a*	Mo *K*α radiation, λ = 0.71075 Å
Hall symbol: -P 2ac 2ab	Cell parameters from 9441 reflections
*a* = 10.400 (1) Å	θ = 1.3–35.6°
*b* = 12.1650 (15) Å	µ = 0.09 mm^−^^1^
*c* = 15.370 (2) Å	*T* = 113 K
*V* = 1944.6 (4) Å^3^	Block, colorless
*Z* = 8	0.32 × 0.30 × 0.28 mm

### Data collection

**Table:**

Rigaku Saturn724 CCD diffractometer	2314 independent reflections
Radiation source: rotating anode	2168 reflections with *I* > 2σ(*I*)
graphite multilayer	*R*_int_ = 0.037
Detector resolution: 14.222 pixels mm^-1^	θ_max_ = 27.9°, θ_min_ = 2.7°
ω scans	*h* = −13→13
Absorption correction: multi-scan (*CrystalClear*-SM Expert; Rigaku/MSC, 2009)	*k* = −16→15
*T*_min_ = 0.971, *T*_max_ = 0.974	*l* = −20→20
21571 measured reflections	

### Refinement

**Table:**

Refinement on *F*^2^	Primary atom site location: structure-invariant direct methods
Least-squares matrix: full	Secondary atom site location: difference Fourier map
*R*[*F*^2^ > 2σ(*F*^2^)] = 0.040	Hydrogen site location: difference Fourier map
*wR*(*F*^2^) = 0.103	H atoms treated by a mixture of independent and constrained refinement
*S* = 1.05	*w* = 1/[σ^2^(*F*_o_^2^) + (0.0569*P*)^2^ + 0.6834*P*] where *P* = (*F*_o_^2^ + 2*F*_c_^2^)/3
2314 reflections	(Δ/σ)_max_ < 0.001
144 parameters	Δρ_max_ = 0.40 e Å^−^^3^
0 restraints	Δρ_min_ = −0.20 e Å^−^^3^

### Special details

**Table:**

Geometry. All e.s.d.'s (except the e.s.d. in the dihedral angle between two l.s. planes) are estimated using the full covariance matrix. The cell e.s.d.'s are taken into account individually in the estimation of e.s.d.'s in distances, angles and torsion angles; correlations between e.s.d.'s in cell parameters are only used when they are defined by crystal symmetry. An approximate (isotropic) treatment of cell e.s.d.'s is used for estimating e.s.d.'s involving l.s. planes.
Refinement. Refinement of *F*^2^ against ALL reflections. The weighted *R*-factor *wR* and goodness of fit *S* are based on *F*^2^, conventional *R*-factors *R* are based on *F*, with *F* set to zero for negative *F*^2^. The threshold expression of *F*^2^ > σ(*F*^2^) is used only for calculating *R*-factors(gt) *etc*. and is not relevant to the choice of reflections for refinement. *R*-factors based on *F*^2^ are statistically about twice as large as those based on *F*, and *R*- factors based on ALL data will be even larger.

### Fractional atomic coordinates and isotropic or equivalent isotropic displacement parameters (Å^2^)

**Table:**

	*x*	*y*	*z*	*U*_iso_*/*U*_eq_	
O1	0.59391 (7)	0.42006 (6)	0.57792 (5)	0.01563 (19)	
N1	0.94264 (9)	0.63850 (8)	0.68372 (6)	0.0171 (2)	
N2	0.80796 (9)	0.69549 (8)	0.57226 (6)	0.0155 (2)	
N3	0.64694 (9)	0.58137 (7)	0.51402 (6)	0.0146 (2)	
C1	0.84778 (10)	0.61467 (9)	0.62723 (7)	0.0137 (2)	
C2	0.78414 (10)	0.51204 (9)	0.62574 (7)	0.0137 (2)	
C3	0.82323 (11)	0.43170 (9)	0.68436 (7)	0.0164 (2)	
H3	0.7818	0.3621	0.6855	0.020*	
C4	0.92347 (11)	0.45420 (9)	0.74123 (7)	0.0185 (2)	
H4	0.9539	0.4001	0.7808	0.022*	
C5	0.97766 (11)	0.55837 (9)	0.73836 (7)	0.0184 (2)	
H5	1.0449	0.5740	0.7783	0.022*	
C6	0.66943 (10)	0.49939 (9)	0.57020 (7)	0.0130 (2)	
C7	0.74197 (10)	0.66576 (8)	0.49201 (7)	0.0134 (2)	
C8	0.83702 (11)	0.62728 (9)	0.42121 (7)	0.0168 (2)	
H8A	0.9065	0.5818	0.4465	0.020*	
H8B	0.7924	0.5845	0.3755	0.020*	
C9	0.88986 (11)	0.73498 (9)	0.38493 (7)	0.0193 (2)	
H9A	0.9265	0.7242	0.3261	0.023*	
H9B	0.9571	0.7656	0.4235	0.023*	
C10	0.77185 (12)	0.81065 (10)	0.38176 (8)	0.0231 (3)	
H10A	0.7316	0.8083	0.3234	0.028*	
H10B	0.7969	0.8875	0.3947	0.028*	
C11	0.67779 (10)	0.76755 (9)	0.45118 (7)	0.0160 (2)	
H11A	0.5946	0.7474	0.4242	0.019*	
H11B	0.6620	0.8244	0.4960	0.019*	
H1	0.8471 (15)	0.7598 (14)	0.5750 (10)	0.030 (4)*	
H2	0.5769 (16)	0.5770 (12)	0.4815 (10)	0.024 (4)*	

### Atomic displacement parameters (Å^2^)

**Table:**

	*U*^11^	*U*^22^	*U*^33^	*U*^12^	*U*^13^	*U*^23^
O1	0.0148 (4)	0.0129 (4)	0.0193 (4)	−0.0014 (3)	0.0001 (3)	0.0009 (3)
N1	0.0166 (4)	0.0201 (5)	0.0147 (4)	−0.0014 (4)	−0.0011 (3)	−0.0010 (4)
N2	0.0177 (5)	0.0121 (5)	0.0166 (4)	−0.0029 (4)	−0.0033 (4)	0.0003 (3)
N3	0.0123 (4)	0.0140 (4)	0.0175 (4)	−0.0023 (3)	−0.0029 (4)	0.0020 (3)
C1	0.0134 (5)	0.0147 (5)	0.0131 (5)	0.0007 (4)	0.0024 (4)	−0.0013 (4)
C2	0.0135 (5)	0.0146 (5)	0.0130 (5)	0.0008 (4)	0.0008 (4)	−0.0010 (4)
C3	0.0185 (5)	0.0146 (5)	0.0161 (5)	0.0016 (4)	0.0010 (4)	−0.0001 (4)
C4	0.0201 (5)	0.0205 (5)	0.0147 (5)	0.0049 (4)	−0.0008 (4)	0.0019 (4)
C5	0.0162 (5)	0.0249 (6)	0.0142 (5)	0.0009 (4)	−0.0018 (4)	−0.0007 (4)
C6	0.0130 (5)	0.0121 (5)	0.0139 (5)	0.0016 (4)	0.0021 (4)	−0.0016 (4)
C7	0.0129 (5)	0.0122 (5)	0.0152 (5)	−0.0014 (4)	−0.0008 (4)	0.0011 (4)
C8	0.0179 (5)	0.0153 (5)	0.0171 (5)	0.0017 (4)	0.0006 (4)	0.0003 (4)
C9	0.0182 (5)	0.0191 (6)	0.0206 (5)	0.0000 (4)	0.0036 (4)	0.0028 (4)
C10	0.0252 (6)	0.0196 (6)	0.0246 (6)	0.0040 (5)	0.0057 (5)	0.0082 (5)
C11	0.0147 (5)	0.0138 (5)	0.0195 (5)	0.0010 (4)	−0.0011 (4)	0.0030 (4)

### Geometric parameters (Å, °)

**Table:**

O1—C6	1.2500 (13)	C4—H4	0.9500
N1—C5	1.3372 (14)	C5—H5	0.9500
N1—C1	1.3458 (14)	C7—C11	1.5404 (14)
N2—C1	1.3609 (14)	C7—C8	1.5428 (15)
N2—C7	1.4571 (13)	C8—C9	1.5262 (16)
N2—H1	0.882 (17)	C8—H8A	0.9900
N3—C6	1.3397 (14)	C8—H8B	0.9900
N3—C7	1.4646 (13)	C9—C10	1.5349 (16)
N3—H2	0.885 (16)	C9—H9A	0.9900
C1—C2	1.4132 (15)	C9—H9B	0.9900
C2—C3	1.3900 (15)	C10—C11	1.5397 (15)
C2—C6	1.4750 (14)	C10—H10A	0.9900
C3—C4	1.3878 (16)	C10—H10B	0.9900
C3—H3	0.9500	C11—H11A	0.9900
C4—C5	1.3876 (16)	C11—H11B	0.9900
			
C5—N1—C1	116.60 (10)	N2—C7—C8	111.75 (9)
C1—N2—C7	119.32 (9)	N3—C7—C8	112.50 (9)
C1—N2—H1	118.1 (10)	C11—C7—C8	103.55 (8)
C7—N2—H1	118.5 (10)	C9—C8—C7	103.17 (9)
C6—N3—C7	123.56 (9)	C9—C8—H8A	111.1
C6—N3—H2	117.6 (9)	C7—C8—H8A	111.1
C7—N3—H2	117.8 (9)	C9—C8—H8B	111.1
N1—C1—N2	117.90 (10)	C7—C8—H8B	111.1
N1—C1—C2	122.96 (10)	H8A—C8—H8B	109.1
N2—C1—C2	119.05 (10)	C8—C9—C10	103.80 (9)
C3—C2—C1	118.28 (10)	C8—C9—H9A	111.0
C3—C2—C6	122.57 (10)	C10—C9—H9A	111.0
C1—C2—C6	118.70 (9)	C8—C9—H9B	111.0
C4—C3—C2	119.29 (10)	C10—C9—H9B	111.0
C4—C3—H3	120.4	H9A—C9—H9B	109.0
C2—C3—H3	120.4	C9—C10—C11	106.36 (9)
C5—C4—C3	117.72 (10)	C9—C10—H10A	110.5
C5—C4—H4	121.1	C11—C10—H10A	110.5
C3—C4—H4	121.1	C9—C10—H10B	110.5
N1—C5—C4	125.11 (10)	C11—C10—H10B	110.5
N1—C5—H5	117.4	H10A—C10—H10B	108.6
C4—C5—H5	117.4	C10—C11—C7	106.30 (9)
O1—C6—N3	121.75 (10)	C10—C11—H11A	110.5
O1—C6—C2	122.26 (10)	C7—C11—H11A	110.5
N3—C6—C2	115.89 (9)	C10—C11—H11B	110.5
N2—C7—N3	107.23 (8)	C7—C11—H11B	110.5
N2—C7—C11	110.45 (9)	H11A—C11—H11B	108.7
N3—C7—C11	111.42 (9)		
			
C5—N1—C1—N2	178.43 (10)	C3—C2—C6—N3	−177.04 (10)
C5—N1—C1—C2	1.72 (16)	C1—C2—C6—N3	10.81 (14)
C7—N2—C1—N1	158.25 (10)	C1—N2—C7—N3	44.94 (13)
C7—N2—C1—C2	−24.91 (15)	C1—N2—C7—C11	166.52 (9)
N1—C1—C2—C3	−1.20 (16)	C1—N2—C7—C8	−78.77 (12)
N2—C1—C2—C3	−177.86 (10)	C6—N3—C7—N2	−40.32 (13)
N1—C1—C2—C6	171.29 (10)	C6—N3—C7—C11	−161.29 (10)
N2—C1—C2—C6	−5.38 (15)	C6—N3—C7—C8	82.94 (12)
C1—C2—C3—C4	−0.71 (16)	N2—C7—C8—C9	−79.95 (10)
C6—C2—C3—C4	−172.89 (10)	N3—C7—C8—C9	159.36 (9)
C2—C3—C4—C5	1.92 (16)	C11—C7—C8—C9	38.94 (10)
C1—N1—C5—C4	−0.39 (17)	C7—C8—C9—C10	−39.83 (11)
C3—C4—C5—N1	−1.43 (17)	C8—C9—C10—C11	25.43 (12)
C7—N3—C6—O1	−169.27 (9)	C9—C10—C11—C7	−1.25 (12)
C7—N3—C6—C2	14.28 (15)	N2—C7—C11—C10	96.69 (10)
C3—C2—C6—O1	6.53 (16)	N3—C7—C11—C10	−144.24 (9)
C1—C2—C6—O1	−165.62 (10)	C8—C7—C11—C10	−23.09 (11)

### Hydrogen-bond geometry (Å, °)

**Table:**

*D*—H···*A*	*D*—H	H···*A*	*D*···*A*	*D*—H···*A*
N2—H1···O1^i^	0.88 (2)	2.05 (2)	2.918 (1)	170 (2)
N3—H2···O1^ii^	0.89 (2)	2.00 (2)	2.876 (1)	172 (1)

Symmetry codes: (i) −*x*+3/2, *y*+1/2, *z*; (ii) −*x*+1, −*y*+1, −*z*+1.

## References

[bb1] Cordeu, L., Cubedo, E., Bandres, E., Rebollo, A., Saenz, X. & Font, N. (2007). *Bioorg. Med. Chem.* **15**, 1659–1669. 10.1016/j.bmc.2006.12.010 17204425

[bb2] Gangjee, A., Vasudevan, A., Queener, S. F. & Kisliuk, R. L. (1996). *J. Med. Chem.* **39**, 1438–1446. 10.1021/jm950786p 8691474

[bb3] Li, J. R., Chen, X., Shi, D. X., Ma, S. L., Li, Q., Zhang, Q. & Tang, J. H. (2009). *Org. Lett* **11**, 1193–1196. 10.1021/ol900093h 19239261

[bb4] Li, J. R., Zhang, L. J., Shi, D. X., Li, Q., Wang, D., Wang, C. X., Zhang, Q., Zhang, L. & Fan, Y. Q. (2008). *Synlett*, pp. 233–236.

[bb5] Ma, S. L., Li, J. R., Zhao, J. M., Zhao, X. F., Yang, X. Q., Zhang, L. J., Wang, L. J. & Zhou, Z. M. (2006). *Tetrahedron*, **62**, 7999–8005.

[bb6] Rigaku/MSC (2009). *CrystalClear-SM Expert* and *CrystalStructure* Rigaku/MSC, The Woodlands, Texas, USA.

[bb7] Robins, R. K. & Hitchings, G. (1958). *J. Am. Chem. Soc.* **80**, 3449–3458.

[bb8] Sheldrick, G. M. (2008). *Acta Cryst.* A**64**, 112–122. 10.1107/S0108767307043930 18156677

[bb9] Zhang, L., Li, J., Shi, D. & Chen, J. (2008). *Acta Cryst.* E**64**, o449. 10.1107/S1600536807066706 PMC2960329 21201476

